# Tumor-Derived Factors Differentially Affect the Recruitment and Plasticity of Neutrophils

**DOI:** 10.3390/cancers13205082

**Published:** 2021-10-11

**Authors:** Ludovica Arpinati, Naomi Kaisar-Iluz, Merav E. Shaul, Christopher Groth, Viktor Umansky, Zvi G. Fridlender

**Affiliations:** 1 Hadassah Medical Center, Institute of Pulmonary Medicine, Faculty of Medicine, Hebrew University of Jerusalem, P.O. Box 12000, Jerusalem 9112001, Israel; ludovica.arpinati@mail.huji.ac.il (L.A.); naomi.kaisar@mail.huji.ac.il (N.K.-I.); meravsha@hadassah.org.il (M.E.S.); 2 German Cancer Research Center (DKFZ), Skin Cancer Unit, 69120 Heidelberg, Germany; christopher.groth@medma.uni-heidelberg.de (C.G.); v.umansky@dkfz.de (V.U.); 3 Department of Dermatology, Venereology and Allergology, University Medical Center Mannheim, Ruprecht-Karl University of Heidelberg, 68167 Mannheim, Germany; 4 Medical Faculty Mannheim, Mannheim Institute for Innate Immunoscience (MI3), University of Heidelberg, 68167 Mannheim, Germany; 5 Department for Immunobiochemistry, Medical Faculty Mannheim, Heidelberg University, 68167 Mannheim, Germany; 6 European Center for Angioscience (ECAS), Medical Faculty Mannheim, Heidelberg University, 68167 Mannheim, Germany

**Keywords:** neutrophils, CXCL1, cell plasticity, cancer immunology, CXCR2 and CXCR4

## Abstract

**Simple Summary:**

Neutrophils are the most abundant type of circulating leukocytes and play an important role in tumor biology. In the context of cancer, the origin of tumor-associated neutrophils (TANs) as well as the interplay between circulating neutrophil subpopulations (NDN and LDN) are still not clear. Our results show for the first time that TANs can originate from both NDN and LDN, with LDN infiltrating at a higher extent than NDN. CXCL1 and CXCL2 chemokines strongly affect NDN and LDN migration, and tumor-conditioned media differently impact their chemotaxis. Moreover, NDN display tumor-induced phenotypical plasticity, transitioning towards a low-density state (LD-NDN). We found that tumor-conditioned media and CXCL1 promote LD-NDN formation, suggesting that multiple factors mediate neutrophils’ plasticity. Newly formed LD-NDN present similar characteristics to LDN in terms of morphology, functional activity, and surface receptor expression, indicating that a portion of LDN could originate from NDN undergoing phenotypical changes.

**Abstract:**

Neutrophils play a key role in cancer biology. In contrast to circulating normal-density neutrophils (NDN), the amount of low-density neutrophils (LDN) significantly increases with tumor progression. The correlation between these neutrophil subpopulations and intratumoral neutrophils (TANs) is still under debate. Using 4T1 (breast) and AB12 (mesothelioma) tumor models, we aimed to elucidate the source of TANs and to assess the mechanisms driving neutrophils’ plasticity in cancer. Both NDN and LDN were found to migrate in response to CXCL1 and CXCL2 exposure, and co-infiltrate the tumor site ex vivo and in vivo, although LDN migration into the tumor was higher than NDN. Tumor-derived factors and chemokines, particularly CXCL1, were found to drive neutrophil phenotypical plasticity, inducing NDN to transition towards a low-density state (LD-NDN). LD-NDN appeared to differ from NDN by displaying a phenotypical profile similar to LDN in terms of nuclear morphology, surface receptor markers, decreased phagocytic abilities, and increased ROS production. Interestingly, all three subpopulations displayed comparable cytotoxic abilities towards tumor cells. Our data suggest that TANs originate from both LDN and NDN, and that a portion of LDN derives from NDN undergoing phenotypical changes. NDN plasticity resulted in a change in surface marker expression and functional activity, gaining characteristics of LDN.

## 1. Introduction

Neutrophils are the most abundant type of circulating leukocytes in the peripheral blood, playing a key role in the innate immune response and clearance of infection, as well as in tumor development and metastatic progression [1,2,3]. Being the first responders at the site of inflammation, neutrophils exert multiple inflammatory functions, such as degranulation, phagocytosis, secretion of inflammatory cytokines, and production of reactive oxygen species (ROS) [1,4].

Circulating neutrophils can be isolated using a discontinuous density gradient. Our studies, as well as studies by others, have shown that in the case of cancer and other inflammatory diseases [5,6,7] neutrophils are present not only in the granulocytic fraction (normal-density neutrophils (NDN)), where the majority of mature neutrophils can be found, but also in the low-density mononuclear cell fraction (low-density neutrophils (LDN)), together with the lymphocytes and monocytes [8,9].

A recent report by Cassatella and Scapini underlined how the widely used definition of HDN (high-density neutrophils) is essentially incorrect, since it refers to neutrophils with a normal and unchanged density [10]. Authors therefore strongly suggest replacing the term HDN with the more appropriate term NDN (normal-density neutrophils). Supported by other recent works that adopted the more precise term NDN [6,11,12], we opted for the use of NDN when discussing neutrophils in the granulocytic fraction in this work.

We have previously shown that the amount and presence of LDN in mice and humans significantly increases with tumor growth; that they consist of two distinct subtypes, namely mature and immature neutrophils [9]; and that the presence of LDN is an important marker of disease progression in cancer patients [13,14,15]. Recent data on NDN and LDN’s functions in cancer suggest that these two subpopulations carry out different cancer-related functions. For example, in tumor-bearing mice, circulating NDN were found to display cytotoxic abilities and to increase tumor cell death [11,16], while LDN appeared to promote tumor development, with a reduced cytotoxicity towards tumor cells, and a stronger suppression of T cell proliferation [9,17]. Although NDN and LDN present different characteristics in terms of specific functions and morphology, there are currently no definite markers that allow for a clear separation between them, rendering the density gradient the most effective way to distinguish them from one another. Furthermore, neutrophils display phenotypical plasticity (reviewed in [18,19]). In mice, a portion of circulating NDN was found in the mononuclear fraction [9].

Neutrophils are rapidly released from the bone marrow (BM) into the circulation. Besides the granulocyte colony stimulating factor (G-CSF), which is a key player in neutrophils’ maturation and release from the BM [20,21], the chemotactic receptors CXCR2 and CXCR4 play an important role in regulating neutrophil homeostasis [22,23,24] by controlling their retention in the BM (via CXCR4) and allowing for neutrophils’ release into the circulation (via CXCR2).

Similarly to NDN and LDN, tumor-associated neutrophils (TANs) were shown to acquire both anti (N1-TANs) and pro-tumor properties (N2-TANs), depending on the cues encountered in the tumor milieu [25,26,27]. This polarization towards an N1 or N2 state is regulated by factors such as Interferon (IFN)-β and the Transforming Growth Factor (TGF)-β [25,28], respectively. However, other cytokines and soluble factors in the tumor microenvironment have been shown to modulate neutrophils’ phenotype and functions, such as S100A8/A9 [29,30] and IL17 [31,32].

Although these neutrophil subtypes (namely NDN, LDN, N1-TAN, and N2-TAN) are present in cancer and are known to be involved in both tumor progression and metastatic spreading, it is still unclear what the link between the circulating neutrophils (NDN and LDN) and their counterparts within the tumor (TANs) is. The exact source of TANs as well as the origin of LDN in the circulation, are still not fully understood. Therefore, the interplay between NDN and LDN requires further investigation.

In the current work, we aimed to unveil the source of TANs by assessing the differential chemotactic forces driving NDN and LDN migration and infiltration into the tumor tissue. We next focused on the relationship between NDN and LDN, investigating the phenotypical changes and the mechanisms behind the transition from NDN to LDN.

## 2. Materials and Methods

### 2.1. Cell Lines

AB12 (lung mesothelioma) and 4T1 (breast cancer) cells were cultured and maintained in DMEM media supplemented with 10% heat-inactivated fetal bovine serum (FBS), 2 mM glutamine, 100 U/mL penicillin, and 100 µg/mL streptomycin (all from Biological Industries). Cell cultures were maintained at 37 °C and 5% CO_2_. The AB12-GCSF cell line was prepared via transfection with the lentiviral vector and subsequent selection by puromycin, as previously described [33]. The efficacy of the transfection was measured by flow cytometry assessing the GFP positive population. GFP^+^ cells were then isolated using a BD FACS Aria II sorter. The 4T1-luciferase transfected cell line was a kind gift from Prof. Z. Granot. The AB12-GCSF and 4T1-luciferase cell lines were maintained in cell culture at the same conditions as the parental cell line. Cultures were regularly tested for mycoplasma contamination.

The Murine 4T1 cell line was purchased from the American Type Culture Collection (ATCC, Manassas, VA, USA). AB12, a murine malignant mesothelioma cell line, derived from an asbestos-induced tumor in a Balb/C mouse, was kindly provided by Prof. Steven Albelda, University of Pennsylvania, PA, USA.

The cell lines were expanded and cryopreserved according to ATCC guidelines.

### 2.2. Animal Models

Five to seven weeks old female Balb/C mice (20–25 g) were purchased from Harlan Laboratories (Jerusalem, Israel). All mice were housed under specific pathogen-free conditions at the Hebrew University School of Medicine Animal Resource Center. Mice were injected subcutaneously in the flank with 1–2 × 10^6^ AB12 tumor cells (parental or GCSF-transfected cell line) or injected orthotopically in the mammary fat pad with 0.5–1 × 10^6^ 4T1 tumor cells. Tumor size was regularly monitored and measured using a calibrated caliper. Tumor volume was calculated using the formula: 6 × (length × width^2^)/3.14. All animals were euthanized prior to surgery. All protocols were approved by the Institutional Animal Care and Use Committee of the Hebrew University School of Medicine.

### 2.3. Murine Neutrophils Isolation

Tumor-bearing mice were sacrificed when primary tumors reached a volume of 500–800 mm^3^. Blood was collected by cardiac puncture using insulin syringes previously rinsed with Heparin (Sigma-Aldrich). Blood was diluted in sterile PBS (Biological Industries) containing 0.5% of bovine serum albumin (BSA), gently loaded at a 1:1 ratio onto a discontinuous Histopaque gradient (Histopaque 1119, Sigma-Aldrich, and 1077 Lymphoprep, StemCell Technology), and then centrifuged at 700× *g* for 30 min at room temperature (RT) with no brake.

The low-density fraction (containing LDN, monocytes, and lymphocytes) was collected from the interphase between the plasma and the 1077 Lymphoprep solution, while the NDN were collected from the interface between the 1077 Lymphoprep and the 1119 Histopaque buffer. Red blood cells were lysed by hypotonic lysis. LDN were further isolated from the low-density fraction by using a negative selection kit (Neutrophils Enrichment Kit, StemCell Technologies). Upon isolation, NDN and LDN purity was assessed by FACS and was consistently found to be >95%.

### 2.4. Tumor Cells-Conditioned Media

The 4T1 or AB12 parental tumor cells were seeded in 24-well plates at a concentration of 1 × 10^6^ cells/mL in RPMI containing 10% FBS, 2 mM glutamine, 100 U/mL penicillin, and 100 µg/mL streptomycin (RPMI 10%), and were cultured for 48 h at 37 °C and 5% CO_2_. Following incubation, tumor media were collected in sterile tubes (Lifegene) and centrifuged at 300× *g* for 6 min to allow for cell debris sedimentation. Supernatants were then transferred into new tubes and stored at −80 °C for further use.

### 2.5. Flow Cytometry

Cells were incubated in FACS buffer (PBS buffer containing 2% FBS, 1 mM EDTA, and 0.1% NaN_2_) with anti CD16/32 antibody (Biogems) for 20 min at 4 °C. Upon blocking, cells were incubated for 45 min at 4 °C with fluorescent-labelled antibodies and kept in the dark. After staining, cells were washed with FACS buffer, centrifuged for 5 min with 300× *g* at 4 °C, resuspended, and filtered for FACS analysis. To detect the murine neutrophil population, cells were stained with APC, PE, or BGViolet450-conjugated anti-mouse Ly6G (clone 1A8, all Biolegend). For surface receptor markers evaluation, cells were stained for CD39-APC, CD39-PE, CD73-APC, CD73-PE (all Biogems), or CD11b-FITC (Biolegend). Data were acquired using an LSR-Fortessa Analyzer. Plot analysis was performed using FlowJo version 10.

### 2.6. CFSE and CellTrace Violet Labelling

NDN and LDN were isolated from the peripheral blood of tumor-bearing mice as described above. NDN were stained for CFSE (Biogems), while LDN were stained for CellTrace Violet (ThermoFisher) according to manufacturer’s instructions. Briefly, cells were washed, resuspended in sterile PBS solution at a concentration of 1–10 × 10^6^ cells/mL, and stained with 5 µM CFSE or 5 µM CellTrace Violet for 20 min at 37 °C. Staining was quenched by adding 4–5 volumes of RPMI 10% media for an additional 5 min at 37 °C. Cells were then centrifuged and resuspended in desired medium. Staining was validated by flow cytometry.

### 2.7. Neutrophils Migration Assay In Vitro–Boyden Chamber

Here, 0.5 × 10^6^ NDN or LDN were seeded in 150 µL of RPMI 10% medium onto 5 µm polycarbonate membrane inserts (Millipore) suited for a 24-well plate. In the lower chamber, 600 µL of either RPMI 10%, AB12 tumor-conditioned medium (AB12-TCM), or 4T1 tumor-conditioned medium (4T1-TCM) were added. To assess the different impacts of the chemokines and soluble factors in the NDN and LDN migration, RPMI 10% was enriched with either CXCL1, CXCL2, CXCL12 (100 ng/mL, all from PeproTech), or G-CSF (20 ng/mL, Filgrastim, TEVA). Following 4 h of incubation at 37 °C, cells in the bottom chamber were harvested and counted. The migration induced by the tumor media or tumor-related soluble factors was compared to the spontaneous diffusion (migration in RPMI 10% only).

### 2.8. Tumor Tissue Infiltration Ex Vivo

In order to test the potential differences between NDN and LDN infiltration into the tumor tissue, 4T1 and AB12 tumors were sliced into 3–4 mm sections and each tumor piece was placed in RPMI 10% medium (2 mL/well) in a 12-well plate. Furthermore, 1, 2, or 3 × 10^6^ CFSE-labelled NDN, and/or 1, 2, or 3 × 10^6^ CellTrace Violet-LDN were added alone or in combination to each well containing the tumor slice for 1–2 h at 37 °C.

Following incubation, tumor slices were collected, washed with sterile PBS, minced, and processed in digestion medium (L15 medium (Sigma-Aldrich) containing DNase I (Roche Diagnostics), as well as Elastase and Collagenase II (both from Worthington)) for 40 min at 37 °C. Cell suspensions were then filtered through a 40 µm filter and centrifuged at 300× *g* for 10 min. Pelleted cells were counted and processed for flow cytometry analysis.

Samples were stained using anti-Ly6G antibody to identify the neutrophils’ population within each tumor slice. The presence of infiltrating CFSE^+^NDN and/or CellTrace Violet^+^LDN was determined within the Ly6G^+^ population. The number of infiltrating neutrophils was calculated based on the number of cells in the sample and was then normalized to 1 × 10^6^ total tumor cells.

### 2.9. Neutrophil Adoptive Transfer (In Vivo Infiltration)

To assess NDN and LDN infiltration into tumor tissues in vivo, CFSE-stained NDN and CellTrace Violet-stained LDN were co-injected (10 × 10^6^ NDN + 10 × 10^6^ LDN) intraperitoneally (IP) into 4T1 or AB12 tumor-bearing mice. At 3 h post-IP injection, mice were sacrificed and the blood, tumor, and lungs were harvested and processed. Briefly, blood was processed to collect the granulocytic fraction (for NDN) and the low-density fraction (containing LDN), as explained above. Lungs and tumors were minced and processed in digestion medium as previously described, followed by RBC lysis (Biological Industries). All samples were then stained for Ly6G and processed for flow cytometry analysis. The presence of the CFSE^+^NDN and/or CellTrace Violet^+^LDN was determined within the Ly6G^+^ population in each blood fraction, in the tumor tissue, or in the lungs. The amount of infiltrating cells and/or cells undergoing plasticity changes were quantified as the percentage of CFSE^+^ or CellTrace Violet^+^ cells within the Ly6G^+^ population in each analyzed sample.

### 2.10. Neutrophils Plasticity Assay In Vitro

Isolated NDN or LDN were incubated in RPMI 10% (7–10 × 10^6^ cells/mL) or in either AB12 or 4T1 tumor-derived conditioned media for 3 h at 37 °C. Samples were then diluted in sterile PBS, gently loaded onto a discontinuous gradient, and centrifuged at 700× *g*, 30 min, RT, at brake 0.

The low-density fraction was collected to assess NDN-to-LD plasticity (LD-NDN), while the high-density granulocytic fraction was harvested to evaluate LDN-to-HD plasticity (HD-LDN). LD-NDN and HD-LDN were resuspended in the appropriate buffer for further use and counted to evaluate neutrophils’ plasticity. The proportion of transitioning cells was normalized to the percentage of the ratio between the number of cells found in the opposite fraction and the amount of cells loaded onto the density gradient.

To assess which factors could affect the switch from NDN to LD-NDN, NDN were incubated in RPMI 10% medium in the absence or presence of CXCL1 (100 ng/mL, Peprotech), TNFα (20 ng/mL, PeproTech), rh-GCSF (20 ng/mL, Filgrastim, TEVA), a small molecule inhibitor of CXCR2 (SB265610; 100 nM, Research and Diagnostics Systems), or anti-TGF-β monoclonal antibody (1D11; 10 µg/mL, BioXCell).

### 2.11. Tumor Cells Killing Assay

The 4T1-luciferase labeled cells (0.5×10^5^/well) were seeded in RPMI 2% FBS on a white 96 flat-bottom-well plate, and cells were let to adhere for 2–4 h at 37 °C and 5% CO_2_. Isolated NDN, LDN, or LD-NDN (0.5 × 10^6^/well) were then added to the tumor cells and co-cultured overnight. Following incubation, supernatants were gently aspirated and cells were lysed for 15 min at RT on an orbital shaker and kept in the dark. Following lysis, the luciferase assay substrate solution (Promega) was added to each well and chemiluminescence was immediately read using a Tecan Spark 10M plate reader. Tumor cell proliferation was calculated as the percentage between the signal intensity in each co-culture sample and the signal intensity of the control (tumor cells alone, considered 100% proliferating cells).

### 2.12. Phagocytosis Assay

Phagocytic activity was evaluated by using the Phagocytosis Assay Kit (Cayman Chemical) according to the manufacturer’s instructions. Briefly, isolated NDN, LDN, or LD-NDN (1 × 10^6^/mL) were incubated in RPMI 10% in the presence of the latex beads-rabbit IgG-FITC complex (1:250) for 45 min at 37 °C. To separate the cells which had phagocytosed the fluorescent beads from those binding the beads on the surface, a Trypan Blue quenching solution was added (1:100) for 1–2 min. Cells were then washed and immediately analyzed by FACS.

### 2.13. Neutrophil ROS Production

Isolated NDN, LDN, or LD-NDN were resuspended at a concentration of 1 × 10^6^ cells/mL in Hank’s balanced salt solution (HBSS) containing 2% FBS. Cells were plated onto a white 96 flat-bottom-well plate and let to adhere for about 20 min. Immediately before reading, a solution containing the HRP reagent (40 U/mL, Sigma-Aldrich) + Luminol (0.5 mM, Sigma-Aldrich) was added to each well. Following the addition of HRP and luminol, PMA (10 nM, Sigma-Aldrich) was added to some wells in order to compare ROS production with and without neutrophils’ activation. The chemiluminescence was read using a Tecan Spark 10M plate reader for 1 h at 37 °C.

### 2.14. Statistical Analysis

All statistical analyses were performed using Graph-Pad Prism software version 8. For comparisons between two groups, a paired or unpaired two-tailed *t*-test was performed considering the Gaussian distribution. Comparisons among the groups were conducted using a one-way ANOVA test with an appropriate follow-up test. Differences were considered statistically significant when *p* < 0.05.

## 3. Results

### 3.1. TANs Originate from Both NDN and LDN

**Figure 1:**
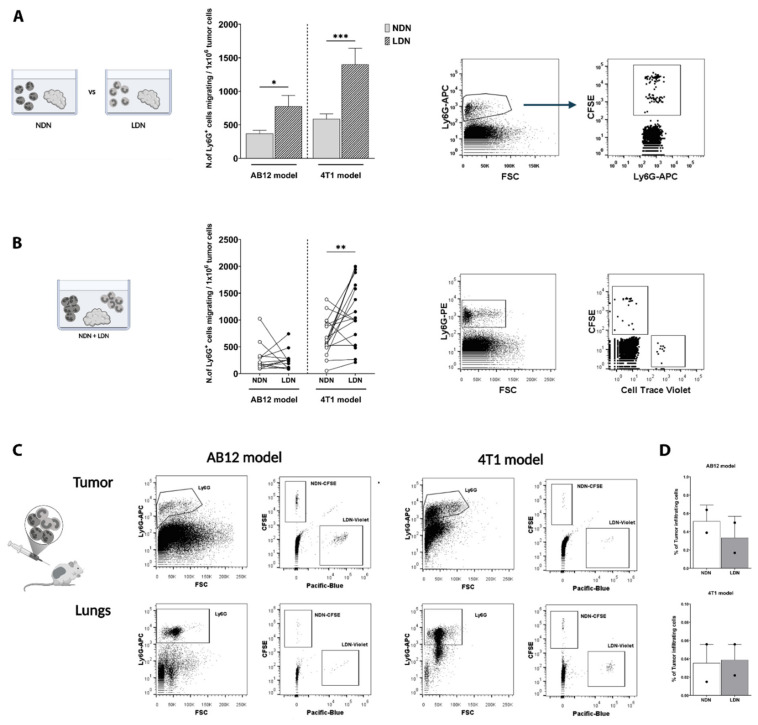
**NDN and LDN co-infiltrate the tumor tissue, with LDN infiltrating to a higher extent than NDN.** (**A**) NDN vs. LDN ex vivo infiltration into the tumor tissue was evaluated in AB12 and 4T1 tumors. A graphic representation of the model (left panel), bar plots showing the quantification of neutrophils’ infiltration (central panel, mean ± SEM, *n* = 13–22), and a representative dot plot with the gating strategy (right panel) are shown. Statistical analysis was performed using a non-paired two-tailed *t*-test: * *p* < 0.05 and *** *p* < 0.001. (**B**) NDN and LDN co-infiltration in the tumor tissue ex vivo. A representation of the co-culture model is shown (left panel). The number of infiltrating NDN and LDN following incubation together with a tumor slice is shown (central panel, *n* = 17–20). Representative dot plots with the gating strategy are provided (right panel). Statistical analysis was performed using a paired two-tailed *t*-test: ** *p* < 0.01. (**C**) IP co-injection of NDN + LDN in vivo. Schematic view of the model (left panel), representative FACS dot plots demonstrating NDN and LDN within the Ly6G^+^ population in the primary tumors (upper plots), and the lungs (bottom plots) in AB12 and 4T1 tumor-bearing mice are shown (right). (**D**) Quantification of co-infiltrating NDN and LDN within the tumor tissue in vivo. The percentage of NDN and LDN infiltration was calculated as the percentage of CFSE ^+^ Ly6G^+^ and CellTrace Violet^+^Ly6G^+^ cells in each tumor following adoptive transfer, wherein *n* = 2 was performed in two separate experiments.

In order to identify which subpopulation of circulating neutrophils (i.e., NDN and/or LDN) represents the source of TANs in the tumor, we first tested the ability of NDN and LDN to infiltrate tumor tissues in an ex vivo model. When culturing tumor tissues ex vivo with either NDN or LDN, we found that both subpopulations were individually able to infiltrate the tumors (Figure 1A). Interestingly, we noted that NDN and LDN infiltration into the tumor was dose-dependent (Figure S1A). However, LDN infiltrated both AB12 and 4T1 tumor models to a greater extent than NDN (Figure 1A). Furthermore, we noted the same trend also at shorter incubation time points (Figure S1B). We did not observe more than 5% of cell death in both NDN and LDN, with up to 4 h of incubation. However, incubation ex vivo overnight resulted in significant neutrophils’ death (not shown), therefore we limited our experiments to 2 h of incubation. We then assessed the interaction between NDN and LDN in their co-infiltration into the tumor tissue. We therefore co-incubated NDN with LDN at a 1:1 ratio in the presence of an AB12 or 4T1 tumor slice following incubation for 2 h. Although NDN and LDN co-infiltrated the tumor site in both models (Figure 1B), we found that in a 4T1 model, LDN infiltrated to a higher extent than NDN (*p* = 0.0075), whereas no differences were detected in AB12. Even when assessing their co-infiltration at shorter time points, LDN resulted in a higher entrance than NDN in a 4T1 model (Figure S1C).

We next evaluated the ability of NDN and LDN to infiltrate tumor tissues in vivo by performing a simultaneous adoptive transfer of fluorescent-labelled NDN and LDN in tumor-bearing mice and assessing their presence in the tumor 3 h later. These in vivo settings confirmed that NDN and LDN can be the source of TANs (Figure 1C). We observed both NDN and LDN not only in the primary tumors of AB12 and 4T1 tumor-bearing mice, but also in other tissues, such as the lungs (Figure 1C). These In vivo settings resulted in a comparable co-infiltration of NDN and LDN in both tumor models (Figure 1D), reflecting our observation following Ex vivo culture with higher amounts of NDN or LDN (Figure S1A).

### 3.2. CXCL1 and CXCL2 Chemokines Drive NDN and LDN Migration

**Figure 2:**
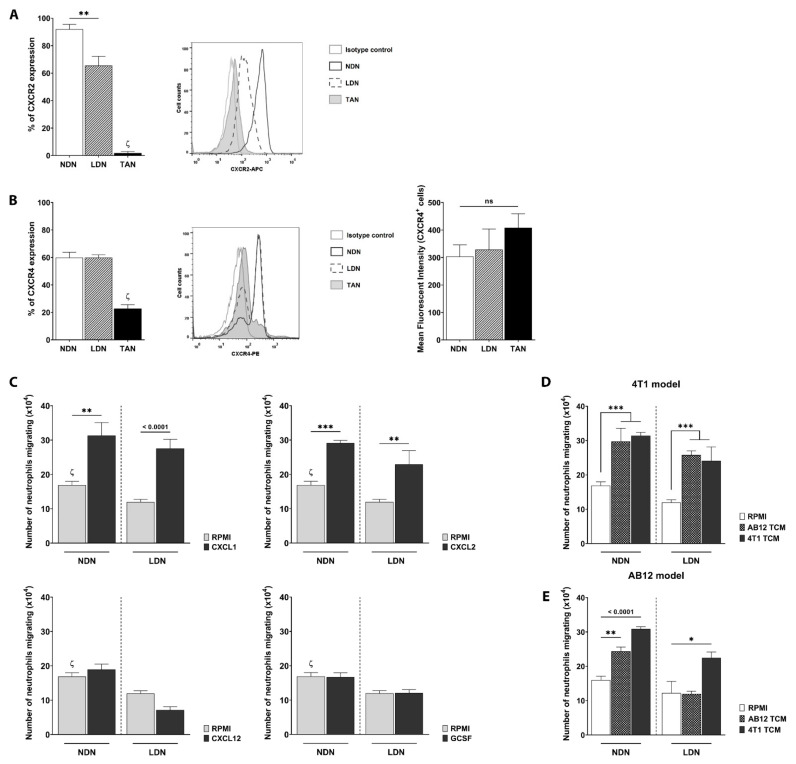
**NDN and LDN migration is strongly induced by tumor-secreted chemokines in vitro.** (**A**) CXCR2 surface expression on NDN, LDN, and TANs in a 4T1 model. Bar plots displaying the percentage of neutrophils expressing the receptor (left panel; mean ± SEM, *n* = 6–7) and a representative FACS histogram (right panel) are shown. (**B**) CXCR4 surface expression on NDN, LDN and TAN in a 4T1 tumor model. Bar plots with the percentage of neutrophils expressing CXCR4 are shown (left panel) (mean ± SEM, *n* = 6–9). A representative histogram (central panel), as well as bar plots showing the quantification of the MFI in the subpopulations (right panel) are presented. In both (**A**,**B**), significant differences between TANs and NDN, and between TANs and LDN are expressed with ζ. Statistical analysis in (**A**,**B**) was performed using a one-way ANOVA with Tukey post-hoc test: ** *p* < 0.01 and ζ *p* < 0.0001. (**C**) Chemokine-induced migration of NDN and LDN in vitro (mean ± SEM, *n* = 3–9). In each graph, significance between NDN and LDN spontaneous migration in RPMI is expressed with a ζ, while significant differences between NDN or LDN spontaneous migration and the chemokine-induced migration are expressed with a star (*). Statistical analysis was performed using an unpaired two-tailed *t*-test: ** and ζ *p* < 0.01; *** *p* < 0.001. (**D**) Evaluation of 4T1-isolated NDN and LDN migration induced by AB12 and 4T1-conditioned media (AB12-TCM and 4T1-TCM, respectively; mean ± SEM, *n* = 3–9). (**E**) NDN and LDN migration induced by AB12-TCM and 4T1-TCM in neutrophils isolated from AB12 tumor-bearing mice (mean ± SEM, *n* = 3). Statistical analysis in (**C**,**D**) was performed using a one-way ANOVA with Dunnett’s post-hoc test: * *p* < 0.05, ** *p* < 0.01, and *** *p* < 0.001.

Focusing on the driving forces behind NDN and LDN recruitment into the tumor, the surface expression of CXCR2 and CXCR4 receptors was checked in NDN, LDN, and TANs. While 91.99 ± 3.5% of NDN and 65.56 ± 6.71% of LDN expressed CXCR2 in a 4T1 tumor model (Figure 2A), only 1.80 ± 1.21% of TANs showed such expression. In contrast, about 60% of both NDN and LDN expressed CXCR4 with similar intensity, and the receptor was found present only on about 20% of TANs (Figure 2B). A similar trend was also observed in NDN, LDN, and TANs isolated from AB12 tumor-bearing mice (Figure S2). Due to this dramatic down-regulation in chemokine receptors’ expression on TANs, we hypothesized that the chemokines binding CXCR2 and CXCR4 (i.e., CXCL1/CXCL2 and CXCL12, respectively) could be responsible for the differential infiltration of NDN and LDN into the tumor.

In an in vitro Boyden chamber assay, we assessed the migratory abilities of 4T1-derived NDN and LDN by using CXCL1, CXCL2, and CXCL12 to stimulate cell migration (Figure 2C). Interestingly, while CXCL1 and CXCL2 strongly induced NDN and LDN migration towards the bottom chamber (Figure 2C, upper panels), CXCL12 did not (Figure 2C, lower left panel). The same trend was observed in neutrophils isolated from AB12 tumor-bearing mice (data not shown). Due to its involvement in neutrophils’ mobilization, we also assessed whether G-CSF would affect NDN and LDN migration. In our hands, G-CSF had no impact on NDN or LDN migration in vitro (Figure 2C, lower right panel).

Considering the differences observed between the tumor models, we proceeded by checking whether 4T1 and AB12-tumor-conditioned media (4T1-TCM and AB12-TCM, respectively) would affect NDN and LDN migration in vitro. NDN and LDN isolated from 4T1 mice showed strong migratory capacities following exposure to both 4T1- and AB12-TCM (Figure 2D), while AB12-derived NDN and LDN appeared to be differently impacted by the two conditioned media (Figure 2E). In fact, although both TCMs were able to drive AB12-NDN migration, only 4T1-TCM had an impact on AB12-LDN (Figure 2E). These results indicate the existence of differences in the predisposition of cancer-related neutrophil subtypes in their migration in different tumor models.

### 3.3. NDN and LDN Phenotypical Plasticity Is Driven by Tumor-Related Factors

**Figure 3:**
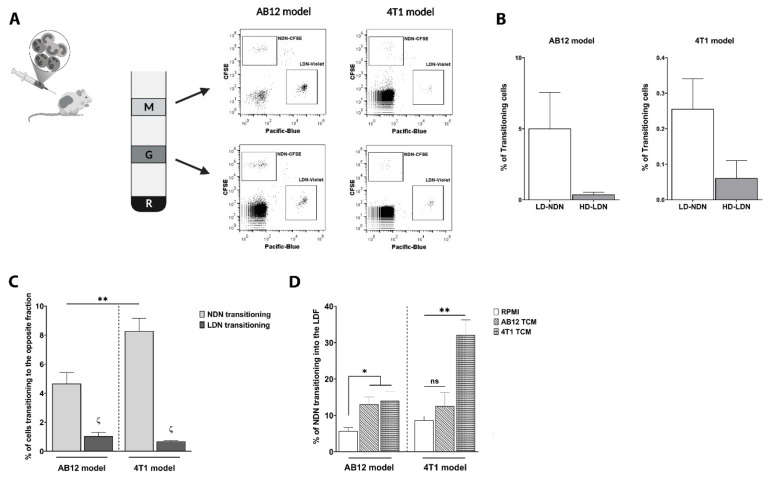
**NDN and LDN display phenotypical plasticity in vivo, strongly induced by 4T1-TCM in vitro.** (**A**) NDN and LDN were co-injected intraperitoneally (IP) in AB12 and 4T1 tumor-bearing mice in vivo. A schematic view of the IP injection model is presented on the left. Representative FACS dot plots of the Ly6G^+^ population in both the monocytic (M) and the granulocytic layer (G) are shown, displaying the co-presence of CFSE^+^NDN and CellTrace Violet^+^LDN in each fraction. (**B**) Quantification of NDN and LDN plasticity following in vivo adoptive transfer in AB12 and 4T1 tumor-bearing mice. Bar plots displaying the percentage of newly formed LD-NDN or HD-LDN in both models are shown (mean ± SEM, *n* = 3). (**C**) Evaluation and comparison between NDN and LDN plasticity in vitro, in both AB12 and 4T1 models (mean ± SEM, *n* = 12–27). Significant differences between NDN and LDN in each model are expressed with a ζ, while differences between AB12-NDN and 4T1-NDN are expressed with a star (*). Statistical analysis was performed using an unpaired two-tailed *t*-test with both ζ and ** *p* < 0.01. (**D**) Effects of AB12 and 4T1-TCM on NDN plasticity in vitro, in AB12 and 4T1 tumor models (mean ± SEM, *n* = 12–25). Statistical analysis was performed using a one-way ANOVA with Dunnett’s post-hoc test: * *p* < 0.05 and ** *p* < 0.01.

Following the adoptive transfer of CFSE^+^NDN and CellTrace Violet^+^LDN to study the source of TANs (Figure 1C), we observed that, besides NDN and LDN infiltration into primary tumors, both neutrophil subtypes underwent phenotypic modulation in the circulation (Figure 3A). By performing a density gradient upon the adoptive transfer of labelled NDN and LDN to separate the circulating low-density fraction (LDF, containing LDN) and the high-density granulocytic fraction (HDF, containing NDN), we found the presence of CFSE^+^NDN in the LDF (Figure 3A, upper panels), as well as CellTrace Violet^+^LDN in the HDF (Figure 3A, lower panels), in both AB12 and 4T1 tumor-bearing mice.

Such phenotypical plasticity has been previously suggested by us and others [9]. Due to the observation that NDN would undergo plasticity changes to a greater extent than LDN (Figure 3B), we decided to characterize the predisposition of each neutrophil subtype to undergo this transition. We therefore isolated circulating neutrophils from both 4T1 and AB12 tumor-bearing mice and assessed NDN or LDN plasticity in vitro by performing a second density gradient following neutrophils’ exposure to different media.

We noted that 4T1-NDN transitioned to a low-density state (LD-NDN) at a significantly higher rate than AB12-NDN (8.61 ± 0.91% vs. 4.66 ± 0.77%, respectively; Figure 3C, light gray bars). Furthermore, the amount of NDN able to undergo plasticity changes was significantly higher than LDN in both models (Figure 3C), with only 1.04 ± 0.27% of AB12-LDN and 0.68 ± 0.05% of 4T1-LDN becoming HD-LDN. We therefore continued the rest of our study by focusing on NDN’s plasticity changes.

Hypothesizing that the differences observed between the two tumor models could be related to a distinct tumor priming, we assessed whether AB12-TCM and 4T1-TCM could impact NDN plasticity. Interestingly, 4T1-TCM was able to induce a massive transition towards an LD-state in both models, markedly increasing the amount of NDN becoming LD-NDN to 14.01 ± 2.51% in AB12-NDN, and to 32.13 ± 4.13% in 4T1-NDN (Figure 3D, RPMI vs. 4T1-TCM). Although AB12-TCM significantly impacted NDN plasticity in the AB12 model, reaching 13.06 ± 2.07% (*p* = 0.035), it did not affect 4T1-derived NDN (Figure 3D, RPMI vs. AB12-TCM). These results are in accordance with our previous observations and allow us to speculate that neutrophils can be differently primed by a specific tumor, resulting in a plasticity change that is dependent on the cancer type.

### 3.4. CXCL1 Induces a Robust Transition from NDN to LD-NDN

**Figure 4:**
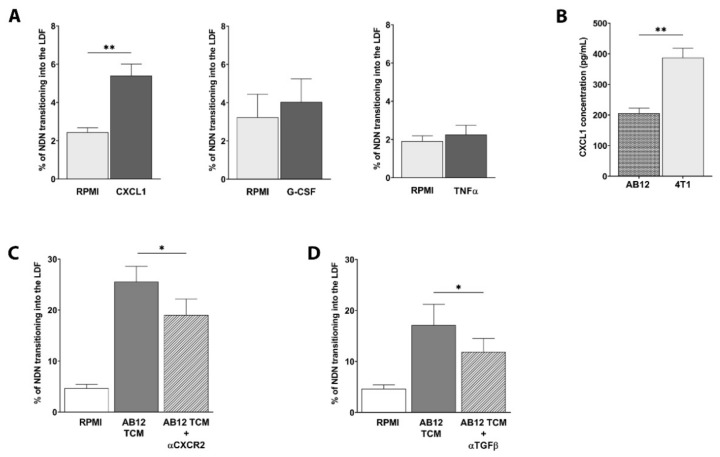
**CXCL1 markedly increases NDN phenotypical switch to LD-NDN in vitro.** (**A**) Phenotypical changes induced by CXCL1, G-CSF, and TNFα (mean ± SEM, *n* = 3–7). (**B**) Production of CXCL1 by tumor cells measured by ELISA (mean ± SEM, *n* = 3). Statistical analysis for (**A**,**B**) was performed using an unpaired two-tailed *t*-test: ** *p* < 0.01. (**C**) Effect of CXCR2 inhibitor on NDN plasticity in AB12-TCM (mean ± SEM, *n* = 3–5). Comparison between AB12-TCM and AB12-TCM + αCXCR2 was performed using a paired two-tailed *t*-test: * *p* < 0.05. (**D**) Effect of TGFβ antibody on NDN plasticity in AB12-TCM (mean ± SEM, *n* = 6). Statistical analysis between AB12-TCM and AB12-TCM + αTGFβ was conducted using a paired two-tailed *t*-test: * *p* < 0.05.

In order to assess which soluble factors secreted by the tumor could drive this plasticity, NDN were exposed to different chemokines (Figure 4A). CXCL1 was able to significantly induce plasticity in AB12-NDN by increasing the amount of LD-NDN from 2.4% to 5.4% (Figure 4A, *p* = 0.0013). In contrast, tumor-related factors such as TNFα and G-CSF had no influence on NDN plasticity in both AB12 (Figure 4A) and 4T1-derived neutrophils (Figure S2). Since CXCL1 had no effect on 4T1-NDN (Figure S2), we quantified the amount of CXCL1 released by AB12 and 4T1 tumor cells via ELISA. Although secreted in both models, CXCL1 levels were significantly higher in 4T1 compared to AB12-conditioned medium (Figure 4B). Therefore, it is possible that NDN isolated from 4T1 tumor-bearing mice may be less sensitive to the direct effect of CXCL1, as they are already primed by such high levels of this chemokine.

In order to validate our observations, we next blocked the CXCR2 receptor on NDN and subsequently evaluated its plasticity shift in tumor-conditioned media. The transition from NDN to LD-NDN was found significantly lower upon the CXCR2 blockade, decreasing from 25.55 ± 3.05% to 19.02 ± 3.17% in AB12-derived neutrophils (Figure 4C, *p* = 0.0332), and from 36.56 ± 8.10% to 16.82 ± 4.18% in 4T1-derived neutrophils (Figure S3, *p* = 0.0484). Since TGFβ is known to be responsible for the phenotypical modulation of cancer-related neutrophils [9,14,25,27], we next assessed its involvement in this plasticity. Blocking TGFβ induced a significant decrease of LD-NDN formation, dropping from 17.12 ± 4.10% to 11.92 ± 2.59% in AB12-derived neutrophils (Figure 4D, *p* = 0.0429), suggesting that multiple tumor-secreted factors are behind neutrophils’ plasticity.

### 3.5. Newly Formed LD-NDN Display Phenotypic Changes and Functional Activity Similar to LDN

**Figure 5:**
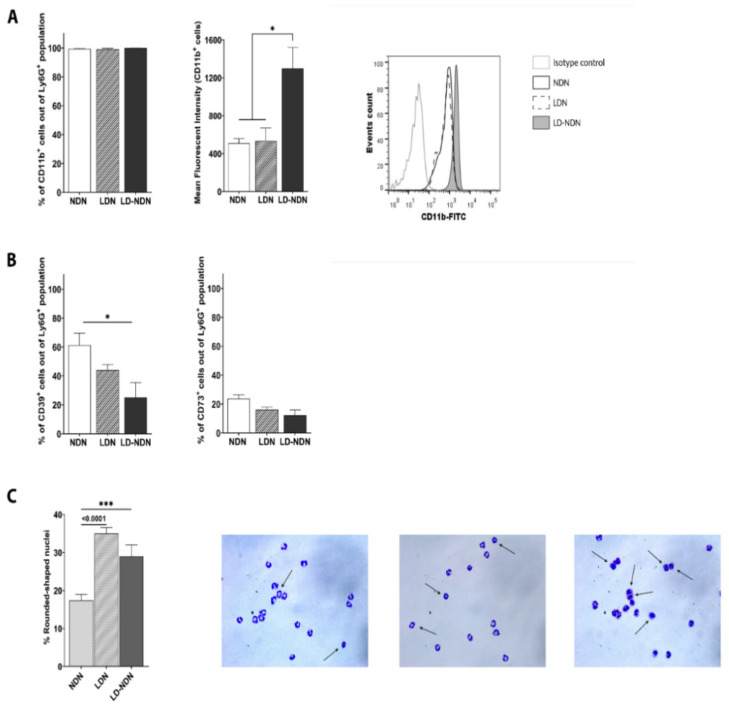
**LD-NDN display a similar profile as LDN in terms of surface receptor markers and nuclear shape.** (**A**) CD11b surface expression on NDN, LDN, and LD-NDN (mean ± SEM, *n* = 5–11). Histograms representing the percentage of Ly6G^+^ cells expressing CD11b, their MFI (left and central panel, respectively), as well as a representative plot showing the increased MFI in LD-NDN (right panel) are presented. (**B**) CD39 and CD73 immunosuppressive marker expression on NDN, LDN, and LD-NDN (mean ± SEM, *n* = 3–7). (**C**) Quantification of immature neutrophils present in NDN, LDN, and LD-NDN. Bar plots show the percentage of immature cells present within each subpopulation (mean ± SEM, *n* = 5–8), and representative pictures of nuclear shapes are presented. Images of NDN, LDN, and LD-NDN’s cytospins with H&E staining (left, center, and right, respectively) are shown, with arrows pointing at the rounded nuclei (magnification 40×). Images were acquired using a Nikon Eclipse e200 microscope and analyzed using ImageJ Software. All statistical analyses (in (**A**–**C**)) were performed using a one-way ANOVA with Tukey post-hoc test: * *p* < 0.05 and *** *p* < 0.001.

We next aimed to characterize the phenotypical and functional consequences of this plasticity in terms of surface marker expression, nuclear morphology, and neutrophilic functions in the newly formed LD-NDN, as compared to NDN and LDN. In order to assess whether this transition could be driven from a degranulation process, we first checked on NDN, LDN, and LD-NDN the surface expression of CD11b, as this integrin is known to be upregulated upon release of gelatinase granules and secretory vesicles from neutrophils [34,35]. We noted that, although all subpopulations expressed CD11b (Figure 5A, left panel), LD-NDN showed a strong increase in MFI compared to NDN and LDN (Figure 5A, central and right panels), supporting the hypothesis that NDN degranulation may lead to a density change.

The surface expression of CD39 and CD73, which have been shown to play a role in the immunosuppressive function of neutrophils [36,37], was then assessed. Both LDN and LD-NDN showed a trend for a reduced expression of CD73 compared to NDN (Figure 5B). Moreover, we detected a significant decrease in the amount of CD39-expressing LD-NDN compared to NDN (25.12 ± 10.36% vs. 61.34 ± 8.28%, respectively; Figure 5B).

We next evaluated neutrophil morphology following plasticity (Figure 5C). Surprisingly, we observed a significant increase in the proportion of immature cells (identified as neutrophils with a rounded nuclear shape) in the LD-NDN subpopulation (28.95 ± 3.08%) compared to NDN (17.44 ± 1.54%), suggesting that both mature and immature neutrophils present in the NDN were prone to undergo this modulation.

**Figure 6:**
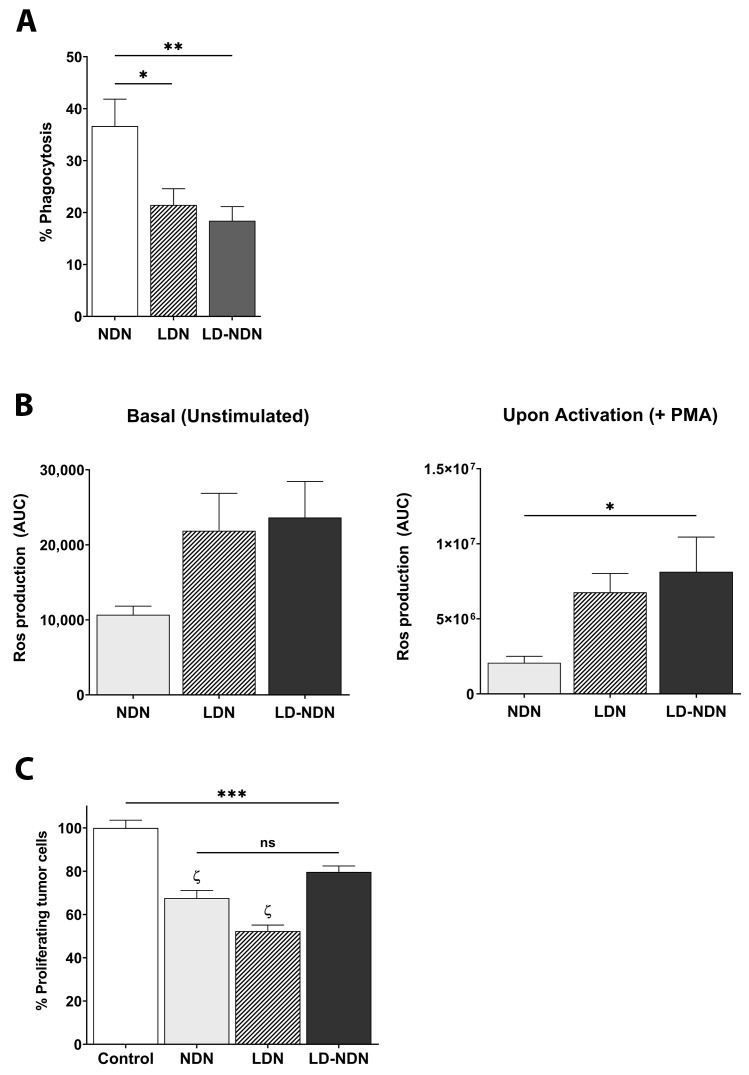
**LD-NDN display functional activity similar to LDN, and different than NDN.** (**A**) Quantification of phagocytosis in NDN, LDN, and LD-NDN (mean ± SEM, *n* = 3). (**B**) ROS production, expressed as area under the curve (AUC), at basal levels (left) and upon PMA stimulation (right) in neutrophil subpopulations (mean ± SEM, *n* = 3–8). (**C**) Evaluation of tumor cell proliferation upon co-culture of 4T1-luciferase cells with NDN, LDN, or LD-NDN. The symbol ζ indicates significant differences between spontaneous tumor cell proliferation (control) and NDN or LDN. Statistical analysis in (**A**–**C**) were performed using a one-way ANOVA with Tukey post-hoc test: * *p* < 0.05, ** *p* < 0.01, *** *p* < 0.001, and ζ *p* < 0.0001.

Due to the phenotypical and morphological similarities observed between LDN and LD-NDN, we next analyzed changes in specific neutrophil-related functions. NDN displayed a significantly higher phagocytic capability than both LDN and LD-NDN (Figure 6A). Moreover, the amount of phagocyting LDN and LD-NDN was found to be comparable (21.48 ± 3.11% and 18.42 ± 2.73%, respectively). We next assessed ROS production in all neutrophil subpopulations. LDN and LD-NDN presented a similar profile, with a trend for a higher basal ROS release than NDN (Figure 6B, left panel, ns). However, following PMA stimulation, LD-NDN released significantly higher levels of ROS compared to NDN (Figure 6B, right panel, *p* = 0.0221).

Finally, neutrophils’ ability to kill tumor cells was evaluated through the quantification of 4T1-luciferase cell proliferation in the presence or absence of NDN, LDN, or LD-NDN. Interestingly, all three subpopulations were found able to significantly decrease 4T1 cell proliferation in a similar manner (Figure 6C).

Taken together, our results show not only how NDN and LDN are the source of TANs, but also that a portion of LDN could originate from NDN undergoing phenotypical changes driven by tumor-related factors.

## 4. Discussion

Neutrophils have been widely described to play a dualistic role in the context of cancer. In fact, while presenting pro-inflammatory and anti-tumor properties [11,28,38,39], they are also able to favor tumor progression and metastatic spreading [17,40,41,42]. As previously mentioned, the presence of a neutrophilic subpopulation within the monocytic fraction upon density gradient in mice and humans (i.e., LDN) has been reported and investigated in the recent years [8,9,14,43]. NDN and LDN (sometimes referred to as PMN-MDSC) were shown to present anti and pro-tumor characteristics, respectively [11,17]. While NDN are formed of mature cells, LDN are composed of both mature and immature neutrophils [9,14]. However, in the recent years, the idea that immature neutrophils are only immunosuppressive, and that mature neutrophils are only cytotoxic towards tumor cells, is under debate [44,45], as studies started to show functional plasticity among cancer-related neutrophils. In our study, in fact, we noticed that this net distinction can no longer be considered, as NDN, for example, were found to express high levels of the immunosuppressive markers CD39 and CD73 [36,46] (Figure 5), and LDN strongly decreased tumor cell proliferation (Figure 6), suggesting that further studies are needed to allow for a full understanding of the role of these different neutrophil subpopulations in cancer.

Among other features, neutrophils retain the ability to rapidly exit the bone marrow and the spleen, reaching the circulation and subsequently extravasating into tissues via chemoattractant gradients and intrinsic forces [47]. It is widely accepted that intratumoral neutrophils (i.e., tumor-associated neutrophils (TAN)) originate from circulating neutrophils. However, the question of whether NDN or LDN (or both) are the source of TANs has not been addressed so far. In the current work, we show for the first time, ex vivo andin vivo, in two different primary tumor models, that TANs can originate from both circulating cancer-related neutrophil subpopulations, namely NDN and LDN.

We showed that LDN have a higher tendency to infiltrate the primary tumor compared to NDN in AB12 and 4T1 models, and that co-culture of NDN and LDN resulted in a higher LDN entrance into 4T1 but not AB12, where the two subpopulations entered at the same extent ex vivo and in vivo (Figure 1 and Figure S1), suggesting that different tumors may influence neutrophil’s infiltration and recruitment into the tumor in a specific way.

Our observations are supported by a recent study by Hsu et al., in which, compared to NDN, LDN were reported to more efficiently accumulate in the livers of mice bearing metastatic lesions [48]. However, while we noted that tumor-derived conditioned media strongly affected both NDN and LDN migration (Figure 2D,E), Hsu et al. reported that media derived from liver-metastatic breast cancer cells only impacted the migration of LDN, but not of NDN [48]. We believe that our contrasting results could be due to the different methods that were used to assess neutrophil migration (i.e., Boyden chamber vs. under-agarose migration assay), as well as due to the different secretions of chemokines and soluble factors in the tumor media used in the studies (i.e., primary tumor vs. metastatic cancer cells-conditioned media). It should be noted that TANs have been shown to be present in the intra and peri-tumoral regions of the primary tumor [49,50], around the metastatic lesions and in the pre-metastatic niches [26], as well as in tumor-draining lymph nodes [51]. Hence, although displaying similar migratory abilities towards tumor-related chemokines (Figure 2), NDN and LDN could be attracted to different tumor compartments, resulting in a differential infiltration of neutrophils’ subpopulations to the diverse tumor areas.

The mechanism behind the differential recruitment of NDN and LDN into the tumor is not fully elucidated, as many factors play a role in neutrophils’ mobilization and chemotaxis (reviewed in [1,52]). However, two chemotactic receptors, namely CXCR4 and CXCR2, have been largely studied in the context of neutrophil migration. In fact, the CXCL12-CXCR4 axis was shown to strongly participate in neutrophils’ mobilization to tissues, as well as in their homing to the bone marrow [24,53]. Similarly, the CXCL1 and CXCL2 chemokines binding to CXCR2 have been shown to be responsible for neutrophil trafficking and migration to the circulation and to inflamed tissues [54,55,56].

In our quest to understand whether these two receptors (namely CXCR2 and CXCR4) differently impact neutrophils’ migration into the tumor, we noted that, in both 4T1 and AB12 models, NDN and LDN presented a high and very similar surface expression of CXCR2 and CXCR4, while TANs expressed significantly lower levels of these receptors, suggesting their possible involvement in the chemoattraction of circulating neutrophils into the tumor (Figure 2A,B, and Figure S2). Similar results have been shown in previous studies [57]. For example, in a melanoma model, it was reported that while TANs did not express CXCR2, BM-neutrophils presented the highest levels of the receptor [54]. The same study further showed that the chemokines binding to CXCR2 (e.g., CXCL1 and CXCL2) were responsible for BM-neutrophils’ extravasation into the circulation and for their further migration into the tumor. Moreover, also in the context of melanoma, we recently showed how the chemokines-binding CXCR2 receptor significantly increased neutrophils’ (described as PMN-MDSC) migration in vitro [58].

When assessing which ligands binding to CXCR2 or CXCR4 would impact the migration of circulating neutrophil subpopulations in vitro, we found that CXCL1 and CXCL2 strongly induced NDN and LDN migration at similar extents, while other chemokines known to be involved in neutrophils’ trafficking (i.e., CXCL12 and G-CSF) did not (Figure 2B). Although 4T1-derived NDN and LDN expressed different levels of CXCR2, their migration towards CXCL1 and CXCL2 chemokines was found to be comparable. It is possible that the in vitro culture conditions may have influenced this outcome, and that a lower concentration of the chemokines could result in detectable differences between these two subpopulations. Moreover, CXCL1 and CXCL2 do not exclusively bind to CXCR2, but also to other chemotactic receptors (e.g., CXCR1). Different expression levels of these receptors on LDN compared to NDN, which were not evaluated in this study, might have affected this cell migration, resulting in similar migratory abilities towards such chemotactic gradients.

Our findings suggest that CXCL1 and CXCL2 are important contributors to the migration of circulating neutrophils into tumors. However, because of the different impact that tumor-conditioned media had on NDN and LDN (Figure 2D,E), we hypothesize that additional factors involved in NDN and LDN trafficking are yet to be described, and that they may differ between tumor models. A deeper characterization of AB12 and 4T1-secreted factors could help identify additional chemokines and/or cytokines involved in this differential recruitment between the two models. Further studies to address these questions are now being planned.

Although neutrophils’ plasticity has been largely discussed, especially in the context of cancer [18,19,59], whether these changes influence the interplay between NDN and LDN has not been fully investigated. Circulating NDN are considered to be mature and terminally differentiated neutrophils, though a phenotypical change in their density has been previously reported in the circulation of late-stage tumor-bearing mice [9], as well as in lung cancer patients [14], where a portion of NDN was found present in the mononuclear fraction. In line with those observations, when performing the NDN and LDN adoptive transfer to study the origin of TANs, we detected a portion of NDN transitioning to a low-density state (LD-NDN), as well as a part of LDN moving to the granulocytic fraction (HD-LDN; Figure 3A). Surprisingly, we observed that NDN would transition towards an LD-state to a much greater extent than the LDN moving towards the granulocytic fraction (Figure 3C). A maturation process of LDN in these ex vivo conditions seems improbable given the time constrain and the restricted culture conditions. The proportion of LDN undergoing plasticity observed in vitro were in line with the in vivo quantifications, i.e., demonstrating a much lower plasticity of LDN compared to NDN (Figure 3B). We therefore believe that while LDN could derive from a premature release of neutrophils from the BM as well as from a fraction of NDN undergoing plasticity changes, the majority of NDN are not the result of a change in LDN.

Considering the strong impact that CXCL1 had on both NDN and LDN migration, and recent evidence pointing towards the idea that chemotactic receptors, such as CXCR1 and CXCR2, may impact other neutrophilic functions apart from chemotaxis [60], we wondered whether these forces may affect the ability of NDN to undergo plasticity changes and become LDN.

Interestingly, we found that the CXCL1-CXCR2 axis is an important regulator of the NDN to LD-NDN transition, whereas factors such as G-CSF or TNFα were not found to be involved in this process (Figure 4). CXCL1 was able to strongly increase the amount of LD-NDN formation in an AB12 model, but did not impact NDN plasticity in 4T1. However, blocking the CXCR2 receptor markedly decreased the amount of newly formed LD-NDN in both AB12- and 4T1-derived NDN. We next found that there were significantly higher levels of CXCL1 in 4T1-TCM compared to AB12-TCM (Figure 4B). We therefore believe that 4T1-NDN could be less sensitive to the chemokine effects, per se, due to their previous exposure to such high levels of CXCL1. Moreover, although 4T1-TCM considerably increased the amount of NDN becoming LD-NDN in both models, the AB12 tumor-conditioned media only increased LD-NDN formation in AB12-NDN, supporting the idea that different tumors prime neutrophils in different ways.

One major question addressed in our study was whether LD-NDN would display phenotypical changes, other than density, compared to NDN. Firstly, we noted that this density change in NDN was possibly related to a degranulation process (Figure 5A). Moreover, we observed a different functional and phenotypical profile in the newly formed LD-NDN, with more similarities to LDN than NDN (Figure 5 and Figure 6). The phagocytosis activity appeared to be significantly lower in both LDN and LD-NDN compared to NDN, while the ROS production of LD-NDN was found significantly higher than that of NDN. Moreover, similarly to LDN, LD-NDN were highly enriched in immature neutrophils (Figure 5C). It is important to mention that, compared to NDN, all three subpopulations presented similar cytotoxic abilities, and were able to significantly decrease tumor cell proliferation (Figure 6C). Furthermore LD-NDN and LDN presented a lower surface expression of CD39 and CD73, which are considered to be immune-suppressive markers [46,61] (Figure 5B), supporting the idea of a functional plasticity in neutrophils’ subpopulations.

As previously stated, while NDN are described as a homogeneous population, LDN have been shown to be composed of both mature and immature neutrophils [9,14,17]. When studying NDN, LDN, and the LD-NDN nuclear shape, we indeed observed a significant higher presence of rounded-shaped nuclei in LDN and LD-NDN compared to NDN, strengthening the idea that immature neutrophils tend to reside in the mononuclear fraction. However, it is important to emphasize that mature neutrophils were largely present in both LDN and LD-NDN subpopulations, and that a part of NDN displayed an immature phenotype (Figure 5C).

Due to these tumor-induced changes of NDN that result in a new subtype of neutrophils, namely LD-NDN, with similar characteristics to LDN, we hypothesize that the differences noted between NDN and LDN in their tumor infiltration could be partially due to the plasticity changes experienced by neutrophils. Since tumor-secreted factors induced NDN plasticity (Figure 5), we can speculate that at least part of the infiltrating NDN (Figure 1) underwent plastic changes (turning into LD-NDN) before entering the tissue. Further studies are needed to deepen our understanding of this LD-NDN subpopulation and their possible preferential infiltration into the tumor tissue, as well as to understand whether they have a unique impact on tumor growth and progression.

Importantly, we did not include human samples in our studies, limiting the possibility to assess the clinical significance of our findings in human cancer patients. For this purpose, further studies using human neutrophils are warranted.

These similarities between LD-NDN and LDN suggest that a portion of LDN could originate from NDN undergoing phenotypical changes induced by the tumor, which go beyond the idea of a change in density per se. Finally, this NDN plasticity is at least partially mediated by the CXCL1-CXCR2 axis and may be differently influenced by the tumor type.

## 5. Conclusions

Our study clearly demonstrates that TANs can originate from both NDN and LDN, probably through CXCR2 and CXCR4 activation, with a mild preference of LDN infiltration compared to NDN. Furthermore, tumor-secreted chemokines and soluble factors differently affect these neutrophil subpopulations’ activity, resulting in changes in tumor infiltration, but also in important phenotypical plasticity. Furthermore, the tumor-induced plasticity of NDN resulted in the formation of a new cell subset, specifically the LD-NDN, with the CXCL1/CXCR2 axis playing an important role in this formation. These newly formed LD-NDN presented a similar profile to LDN, suggesting that cancer-related LDN could partially originate from NDN undergoing tumor-induced phenotypical changes. Our findings add another layer to the understanding of the complex role and functions of neutrophils in cancer.

## Data Availability

The data presented in this study are available upon request from the corresponding author.

## Supplementary Materials

The following are available online at https://www.mdpi.com/article/10.3390/cancers13205082/s1, Figure S1: Competitive vs. non-competitive NDN and LDN tumor infiltration ex vivo in a 4T1 model; Figure S2: CXCR2 and CXCR4 expression levels on neutrophil subpopulations in the AB12 model; Figure S3: CXCL1, G-CSF, and TNFα do not impact NDN transition towards an LD-state in the 4T1 model; and Figure S4: Blocking CXCR2 receptor in 4T1-TCM significantly reduces the amount of LD-NDN found in the LD in 4T1-derived neutrophils.
