# Supplementary material for: Tumor-Derived Factors Differentially Affect the Recruitment and Plasticity of Neutrophils
Source: Cancers (Basel). 2021 Oct 11;13(20):5082. doi: 10.3390/cancers13205082 (PMC8534125; doi:10.3390/cancers13205082)
Supplement: Supplementary file 1 [file cancers-13-05082-s001.zip › Supplementary Figures Legends_ProofRead final_11.10.21.pdf]

SUPPLEMENTARY MATERIALS

SUPPLEMENTARY FIGURE LEGENDS

Supplementary Figure 1. Competitive vs non-competitive NDN and LDN tumor infiltration ex vivo in a 4T1 model. (**A**) Dose-response of NDN vs LDN ex vivo infiltration after 2 hours of incubation in the presence of 4T1 tumor slices. Bar plots showing the quantification of neutrophils' infiltration (mean ± SEM, $n = 4\text{-}17$) are presented. 1M, 2M, and 3M indicate the amount of NDN or LDN incubated with the tumor, 1×10^6, 2×10^6, or 3×10^6 respectively. (**B**) 2×10^6 NDN or 2×10^6 LDN were incubated in the presence of a 4T1 tumor slice for 1- or 2-hours. The quantification of NDN vs LDN tumor infiltration are shown (mean ± SEM, $n = 6\text{-}17$). Each dot represents one sample. Statistical analysis in **A** and **B** was performed using a non-paired two-tailed *t*-test, *** $p < 0.001$. (**C**) 2×10^6 NDN + 2×10^6 LDN co-infiltration in the tumor tissue ex vivo following 1- or 2-hours incubation). The number of infiltrating NDN and LDN following incubation together with a tumor slice is shown. Statistical analysis was performed using a paired two-tailed *t*-test, * $p <0.05$, ** $p < 0.01$.

Supplementary Figure 2. CXCR2 and CXCR4 expression levels on neutrophil subpopulations in the AB12 model. CXCR2 and CXCR4 surface expression on NDN, LDN and TANs derived from AB12 tumor-bearing mice (mean ± SEM, $n = 7\text{-}16$). Significant differences between NDN and LDN are expressed with a star (i.e., *), while the comparison between TANs and NDN, or TANs and LDN is expressed with ζ. Statistical analysis was performed using a one-way ANOVA with Tukey post-hoc test, *** $p < 0.001$, and $\zeta = p < 0.0001$.

Supplementary Figure 3. CXCL1, G-CSF and TNFα do not impact NDN transition towards an LD-state in the 4T1 model. Phenotypical changes induced by CXCL1, G-CSF and TNFα on the plasticity of 4T1-derived NDN (mean ± SEM, $n = 5\text{-}12$). Statistical analyses were performed using an unpaired two-tailed *t*-test, * $p < 0.05$.

Supplementary Figure 4. Blocking CXCR2 receptor in 4T1-TCM significantly reduces the amount of LD-NDN found in the LD in 4T1-derived neutrophils. Effect of CXCR2 inhibitor in 4T1-TCM on NDN plasticity (mean ± SEM, $n = 3\text{-}6$). Comparison between 4T1-TCM and 4T1-TCM+αCXCR2 was performed using a paired two-tailed *t*-test, * $p < 0.05$.
