# Supplementary figures and images for: Tumor-Derived Factors Differentially Affect the Recruitment and Plasticity of Neutrophils
Source: Cancers (Basel). 2021 Oct 11;13(20):5082. doi: 10.3390/cancers13205082 (PMC8534125; doi:10.3390/cancers13205082)

**A**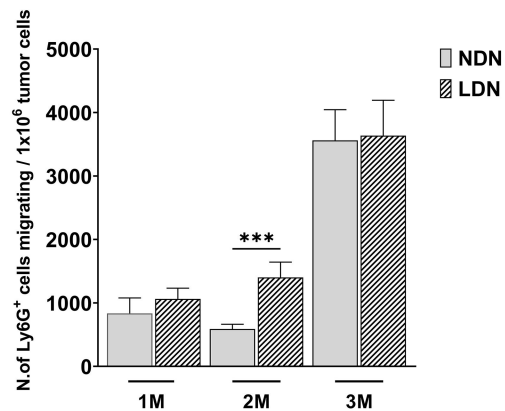**B**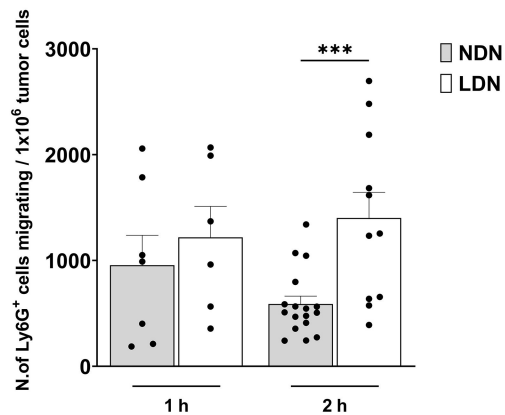**C**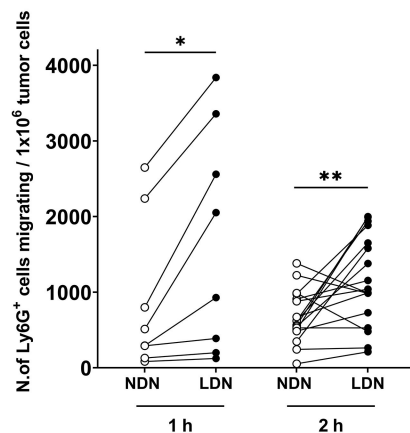

Supplement: Supplementary file 1 [file cancers-13-05082-s001.zip › Supplementary figure 1.pdf]

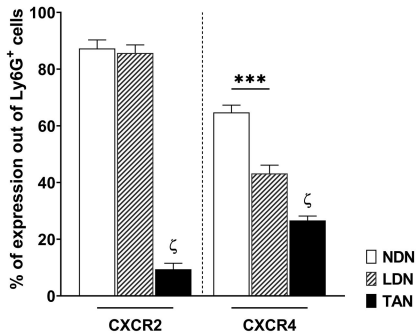

Supplement: Supplementary file 1 [file cancers-13-05082-s001.zip › Supplementary figure 2.pdf]

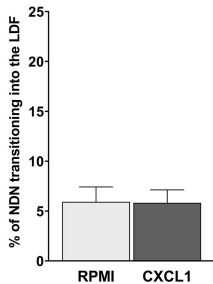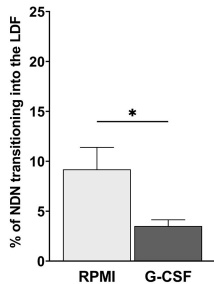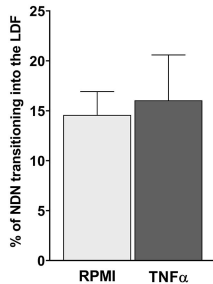

Supplement: Supplementary file 1 [file cancers-13-05082-s001.zip › Supplementary figure 3.pdf]

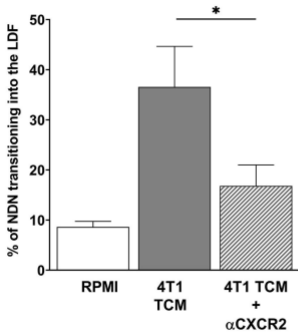

Supplement: Supplementary file 1 [file cancers-13-05082-s001.zip › Supplementary figure 4.pdf]
